# Toll-Like Receptor-4 Is Involved in Mediating Intestinal and Extra-Intestinal Inflammation in *Campylobacter coli*-Infected Secondary Abiotic IL-10^−/−^ Mice

**DOI:** 10.3390/microorganisms8121882

**Published:** 2020-11-27

**Authors:** Sigri Kløve, Claudia Genger, Dennis Weschka, Soraya Mousavi, Stefan Bereswill, Markus M. Heimesaat

**Affiliations:** Institute of Micro Biology, Infectious Diseases and Immunology Charité—University Medicine Berlin, Humboldt-Universität zu Berlin, and Berlin Institute of Health, 12203 Berlin, Germany; sigri.klove@charite.de (S.K.); claudia.genger@charite.de (C.G.); dennis.weschka@charite.de (D.W.); soraya.mousavi@charite.de (S.M.); stefan.bereswill@charite.de (S.B.)

**Keywords:** Toll-like receptor-4, lipooligosaccharide, *Campylobacter coli*, campylobacteriosis model, host-pathogen interaction, secondary abiotic IL-10^−/−^ mice, pro-inflammatory immune responses, intestinal immunopathology, extra-intestinal immune responses

## Abstract

Human *Campylobacter* infections are emerging worldwide and constitute significant health burdens. We recently showed that the immunopathological sequelae in *Campylobacter jejuni*-infected mice were due to Toll-like receptor (TLR)-4 dependent immune responses induced by bacterial lipooligosaccharide (LOS). Information regarding the molecular mechanisms underlying *Campylobacter coli*-host interactions are scarce, however. Therefore, we analyzed *C. coli*-induced campylobacteriosis in secondary abiotic IL-10^−/−^ mice with and without TLR4. Mice were infected perorally with a human *C. coli* isolate or with a murine commensal *Escherichia coli* as apathogenic, non-invasive control. Independent from TLR4, *C. coli* and *E. coli* stably colonized the gastrointestinal tract, but only *C. coli* induced clinical signs of campylobacteriosis. TLR4^−/−^ IL-10^−/−^ mice, however, displayed less frequently fecal blood and less distinct histopathological and apoptotic sequelae in the colon versus IL-10^−/−^ counterparts on day 28 following *C. coli* infection. Furthermore, *C. coli*-induced colonic immune cell responses were less pronounced in TLR4^−/−^ IL-10^−/−^ as compared to IL-10^−/−^ mice and accompanied by lower pro-inflammatory mediator concentrations in the intestines and the liver of the former versus the latter. In conclusion, our study provides evidence that TLR4 is involved in mediating *C. coli*-LOS-induced immune responses in intestinal and extra-intestinal compartments during murine campylobacteriosis.

## 1. Introduction

*Campylobacter* infections are among the most prevalent causes of bacterial infectious gastroenteritis worldwide [1,2]. In most cases, *C. jejuni* and, less frequently, *C. coli* induce the diarrheal disease complex campylobacteriosis in infected human patients. The acute phase of campylobacteriosis is characterized by diarrhea and abdominal cramps, commonly accompanied by fever and bloody stools [3,4]. The disease is mostly self-limiting, and antimicrobial therapy is, therefore, only needed in severe incidents, particularly in infected immunocompromised individuals. In rare cases, however, campylobacteriosis is associated with long-term post-infectious sequelae, such as the autoimmune diseases Guillain–Barré syndrome (GBS), reactive arthritis, or intestinal inflammatory morbidities, including inflammatory bowel disease, coeliac disease, and irritable bowel syndrome [4,5,6]. The majority of campylobacteriosis outbreaks are food-borne due to the fact that *Campylobacter* species reside as commensals in the gut microbiota of several domestic and wild animals [6,7]. Besides contaminated water, raw or undercooked meat from livestock, such as poultry, cattle, pigs, and sheep, are common sources of *C. jejuni* and *C. coli* transmission to humans [8,9]. Although *C. jejuni* and *C. coli* share many reservoirs, their prevalence rates differ greatly. In contaminated sheep and pig meat, for instance, most *Campylobacter* isolates have been identified as *C. coli* [8,10]. Even though *C. coli* causes far fewer infections than *C. jejuni*, its health burden is still considerable. Depending on the geographical region, *C. coli* can account for up to 25% of reported *Campylobacter* infections [11]. Data collected in the framework of a population-based sentinel surveillance scheme for *Campylobacter* infections in the United Kingdom revealed, for instance, that *C. coli* was the causative agent in 25,000 gastroenteritis cases with 11 lethal outcomes [12].

The innate host immune system is pivotal in combating bacterial infections [13]. Whereas the accurate spatial and temporal regulation of the innate immune response is crucial for the host to successfully eliminate the pathogen, overamplification or dysregulation of the pathogen-induced inflammation results in disrupted intestinal homeostasis and, consequently, the development of immunopathology [14]. Under certain circumstances, Gram-negative bacterial infections may result in sepsis with the lethal outcome due to an uncontrolled release of pro-inflammatory mediators following activation of the innate immune receptor Toll-like receptor (TLR)-4 by lipopolysaccharide (LPS) or lipooligosaccharide (LOS), both constituting major cell wall components of Gram-negative bacteria [15]. We and others have previously shown that TLR4 signaling induced by LOS is essential for the intestinal and extra-intestinal, including systemic, immunopathological host responses following murine *C. jejuni* infection [16,17,18,19,20]. Clinical studies confirmed the important role of bacterial LOS in intestinal pathogenesis and that modification of LOS increases the pathogenic potential of *C. jejuni* [21]. In particular, LOS sialylation is associated with severe gastroenteritis and supports the induction and progression of severe forms of campylobacteriosis, including the development of post-infectious sequelae. Both LPS and LOS consist of a core oligosaccharide and a lipid A component, but LOS lacks the prolonged O-antigens found in LPS [22]. Lipid A, however, is responsible for most of the immunostimulatory effects of LPS and LOS [23]. Despite the significance of LOS in the pathogenicity of campylobacteriosis, only very little is known about the genetic and phenotypic diversity of *C. coli* LOS and its pathogenic properties in intestinal infection [24]. Recent studies revealed a high genetic diversity of LOS among *C. coli* strains isolated from different sources, assigning the LOS to 12 different genetic subtypes [24,25,26]. Furthermore, species-specific phenotypic features of *C. coli* could be identified, which might possibly explain differences between *C. jejuni* and *C. coli* in terms of host adaptation [24].

As a result of the predominance of *C. jejuni* in human campylobacteriosis, research on *C. coli* has largely been neglected [12]. Moreover, the clinical symptoms induced by *C. coli* are considered to be indistinguishable from those following *C. jejuni* infection [11]. Given that our knowledge of mechanisms underlying *C. coli*-host interactions is rather scarce, we here investigated *C. coli*-induced campylobacteriosis in an established clinical murine infection model. Our group recently showed that upon microbiota depletion by broad-spectrum antibiotic treatment, secondary abiotic IL-10^−/−^ mice could not only be stably colonized by *C. jejuni* following peroral challenge but also presented with key features of acute campylobacteriosis seen in humans, such as wasting and bloody diarrhea within one week [27]. The underlying mechanisms for these severe *C. jejuni*-induced immunopathological responses mounting in acute ulcerative enterocolitis were the abrogated colonization resistance following microbiota depletion, facilitating the establishment of the pathogen in the host’s gastrointestinal tract, and furthermore, the lack of IL-10, enhancing the susceptibility of mice to *C. jejuni* LOS [27]. Therefore, microbiota-depleted and *C. jejuni*-infected IL-10^−/−^ mice display pronounced TLR4-dependent LOS-induced innate and adaptive immune responses that are not restricted to the intestinal tract but can also be observed in extra-intestinal, including systemic, compartments [17,28,29,30,31,32,33,34,35]. Very recently, we were able to show that TLR4-deficient IL-10^−/−^ mice carrying a human gut microbiota displayed less distinct immune responses upon peroral *C. coli* infection as compared to IL-10^−/−^ counterparts [36].

In order to investigate *C. coli*-induced campylobacteriosis and to determine the immunopathogenic role of *C. coli* LOS in the absence of any commensal gut microbiota, we here assessed the gastrointestinal colonization properties and the clinical, macroscopic, and microscopic inflammatory sequelae in intestinal and extra-intestinal compartments following peroral challenge of secondary abiotic TLR4-deficient IL-10^−/−^ mice.

## 2. Materials and Methods

### 2.1. Ethics Statement

The mouse studies were approved by the local commission for animal experiments (“Landesamt für Gesundheit und Soziales”, LaGeSo, Berlin, Germany, registration numbers G0172/16 and G0247/16) and performed according to the European Guidelines for animal welfare (2010/63/EU). Throughout the experiment, the clinical conditions of mice were evaluated twice daily.

### 2.2. Generation of Secondary Abiotic Mice

IL-10^−/−^ and TLR4^−/−^ IL-10^−/−^ mice (in C57BL/10 background) were raised under specific pathogen-free conditions in the identical room within the Forschungseinrichtungen für Experimentelle Medizin (FEM, Charité—University Medicine Berlin, Berlin, Germany). Mice were maintained in cages covered with filter tops under standard conditions (12 h light/12 dark cycle, 55 ± 15% humidity, 22–24 °C room temperature), had unlimited access to autoclaved standard chow (ssniff R/M-H, V1534-300, Sniff, Soest, Germany), and were handled under aseptic conditions inside an experimental intermediate barrier level. In order to guarantee stable gastrointestinal *C. coli* or *E. coli* colonization [16], the physiological murine colonization resistance was abrogated by antibiotic microbiota depletion, as described earlier [16,37]. In brief, immediately after weaning, 3-week-old mice were subjected to an eight-week course of broad-spectrum antibiotic treatment by adding ampicillin plus sulbactam (1 g/L; Ratiopharm, Ulm, Germany), vancomycin (500 mg/L; Cell Pharm, Hannover, Germany), ciprofloxacin (200 mg/L; Bayer Vital, Leverkusen, Germany), imipenem (250 mg/L; Fresenius Kabi, Bad Homburg, Germany), and metronidazole (1 g/L; B. Braun, Melsungen, Germany) to the autoclaved drinking water (*ad libitum*) [37]. To assure antibiotic irrigation, the antibiotic cocktail was replaced by autoclaved drinking water three days prior to bacterial gavage of secondary abiotic mice.

### 2.3. Bacterial Challenge and Gastrointestinal Colonization and Translocation

The used *C. coli* strain had originally been isolated from the stool of a patient suffering from bloody diarrhea and was kindly provided by Dr. Torsten Semmler (Robert-Koch-Institute, Berlin, Germany). A commensal intestinal *Escherichia coli* isolate from a healthy wildtype mouse served as a Gram-negative rod control [38,39]. The absence of commonly known virulence factors of pathogenic *E. coli*, such as *stx* 1 and 2, *catA*, *hlyA*, *cspA*, *katP*, and *astA*, was verified by PCR analysis [39].

On two consecutive days (i.e., days 0 and 1), sex- and age-matched secondary abiotic mice (three months of age) were perorally challenged with 10^9^ colony forming units (CFU) of the *C. coli* patient isolate or the commensal *E. coli* strain by gavage. Throughout the experiment, mice were maintained in a sterile environment (autoclaved food and drinking water) and handled under strict aseptic conditions to prevent contamination.

To monitor the colonization properties over a 28-day-period, *C. coli* and *E. coli* loads were enumerated in fecal samples collected on selected days post-infection (p.i.) and in luminal samples derived from distinct parts of the gastrointestinal tract (i.e., from the stomach, duodenum, ileum, and colon) upon necropsy by culture, as stated elsewhere [16,40]. In order to quantity *C. coli* burdens, samples were homogenized with a sterile pistil and serial dilutions plated onto Columbia agar plates containing 5% sheep blood and, additionally, onto selective Karmali plates (both from Oxoid, Wesel, Germany). The inoculated plates were incubated in a jar under microaerophilic conditions for 48 h at 37 °C. *E. coli* loads were enumerated in serial dilutions of homogenized fecal samples plated onto Columbia agar plates containing 5% sheep blood and selective MacConkey agar plates (Oxoid, Wesel, Germany) following incubation for 48 h at 37 °C in aerobic atmosphere. Bacterial translocation of the respective bacterial strains was assessed in homogenized ex vivo biopsies obtained from mesenteric lymph nodes (MLN), spleen, kidneys, liver, and lungs and from cardiac blood, as described elsewhere [16,40]. The detection limit of viable pathogens was ≈ 100 CFU per g (CFU/g).

### 2.4. Clinical Conditions

Before and after bacterial application, the clinical conditions of mice were evaluated by daily applying a standardized clinical score (maximum 12 points). The score addressed the clinical aspect/wasting (0: normal; 1: ruffled fur; 2: less locomotion; 3: isolation; 4: severely compromised locomotion, pre-final aspect), the abundance of blood in feces (0: no blood; 2: microscopic detection of blood by the Guajac method using Hemoccult, Beckman Coulter, Krefeld, Germany; 4: macroscopic blood visible), and diarrhea (0: formed feces; 2: pasty feces; 4: liquid feces), as described earlier [33]. Fecal blood positivity rates were determined by the ratio of overt and occult fecal blood positive mice to the total number of analyzed animals.

### 2.5. Sampling Procedures

On day 28 p.i., mice were sacrificed by CO_2_ asphyxiation. Immediately after, ex vivo biopsies from spleen, kidneys, liver, lungs, and MLN, as well as luminal gastrointestinal samples (from the stomach, duodenum, ileum, and colon), were isolated under sterile conditions. Cardiac blood was obtained for serum cytokine measurements. Colon, MLN, and liver tissue samples were collected from each mouse for subsequent immunological, histological, and microbiological analyses.

### 2.6. Histopathology

Histological analyses were performed in colonic ex vivo biopsies following immediate fixation in 5% formalin and embedding in paraffin. Sections (5 μm) were stained with hematoxylin and eosin (H&E), examined by light microscopy (100× magnification), and histopathological changes in the large intestines were quantitatively assessed by applying an established histopathological scoring system, as reported previously [41]: Score 1: minimal inflammatory cell infiltrates in the mucosa with intact epithelium. Score 2: mild inflammatory cell infiltrates in the mucosa and submucosa with mild hyperplasia and mild goblet cell loss. Score 3: moderate inflammatory cell infiltrates in the mucosa with moderate goblet cell loss. Score 4: marked inflammatory cell infiltration into the mucosa and submucosa with marked goblet cell loss, multiple crypt abscesses, and crypt loss.

### 2.7. In Situ Immunohistochemistry

The distinct immune cell population was quantified in colonic paraffin sections by applying in situ immunohistochemistry, as described earlier [42,43,44,45]. In brief, apoptotic epithelial cells, macrophages/monocytes, T lymphocytes, regulatory T cells (Tregs), and B lymphocytes were detected in 5 μm colonic paraffin sections stained with primary antibodies directed against cleaved caspase 3 (Asp175, Cell Signaling, Beverly, MA, USA, 1:200), F4/80 (# 14-4801, clone BM8, eBioscience, San Diego, CA, USA, 1:50), CD3 (#N1580, Dako, 1:10), FOXP3 (clone FJK-165, #14-5773, eBioscience, 1:100), and B220 (No. 14-0452-81, eBioscience; 1:200), respectively. Positively stained cells were then examined by light microscopy (magnification 100× and 400×), and for each mouse, the average number of respective positively stained cells was determined within at least six high power fields (HPF, 0.287 mm^2^, 400× magnification) by a blinded investigator.

### 2.8. Pro-Inflammatory Mediator Measurements in Intestinal, Extra-Intestinal, and Systemic Compartments

Ex vivo biopsies obtained from the colon (longitudinally cut strips of approximately 1 cm^2^), MLN (3 lymph nodes), and liver (approximately 1 cm^3^) were washed in phosphate-buffered saline (PBS; Thermo Fisher Scientific, Waltham, MA, USA) and placed into 24-flat-bottom well culture plates (Thermo Fisher Scientific, Waltham, MA, USA) containing 500 μL serum-free RPMI 1640 medium (Thermo Fisher Scientific, Waltham, MA, USA) supplemented with penicillin (100 µg/mL) and streptomycin (100 µg/mL; Biochrom, Berlin, Germany). After incubation at 37 °C for 18 h, respective culture supernatants, as well as serum samples, were tested for interferon-γ (IFN-γ) and tumor necrosis factor-α (TNF-α) by the Mouse Inflammation Cytometric Bead Assay (CBA; BD Biosciences, Heidelberg, Germany) in a BD FACSCanto II flow cytometer (BD Biosciences, Heidelberg, Germany). Nitric oxide (NO) was measured by the Griess reaction, as described elsewhere [37].

### 2.9. Statistical Analysis

Medians and levels of significance were determined with GraphPad Prism v8, USA. The Student’s t-test was used for pairwise comparisons of normally distributed data, and the Mann–Whitney test was used for pairwise comparisons of not normally distributed data. For multiple comparisons, the one-sided ANOVA with Tukey post-correction was used for normally distributed data, and the Kruskal–Wallis test with Dunn’s post-correction for not normally distributed data. Two-sided probability *p*-values ≤ 0.05 were considered significant. Data were pooled from three independent experiments with the following cohort sizes per individual experiment: IL-10^−/−^ mice +*E. coli* (4,3,3); TLR4^−/−^ IL-10^−/−^ mice +*E. coli* (5,4,4); IL-10^−/−^ mice +*C. coli* (6,5,5); TLR4^−/−^ IL-10^−/−^ mice +*C. coli* (8,7,7). Definite outliers were removed after identification by using Grubb’s test (α = 0.001).

## 3. Results

### 3.1. Role of TLR4 in Intestinal Colonization of C. coli and E. coli in Secondary Abiotic IL-10-/- Mice

**Figure 1:**
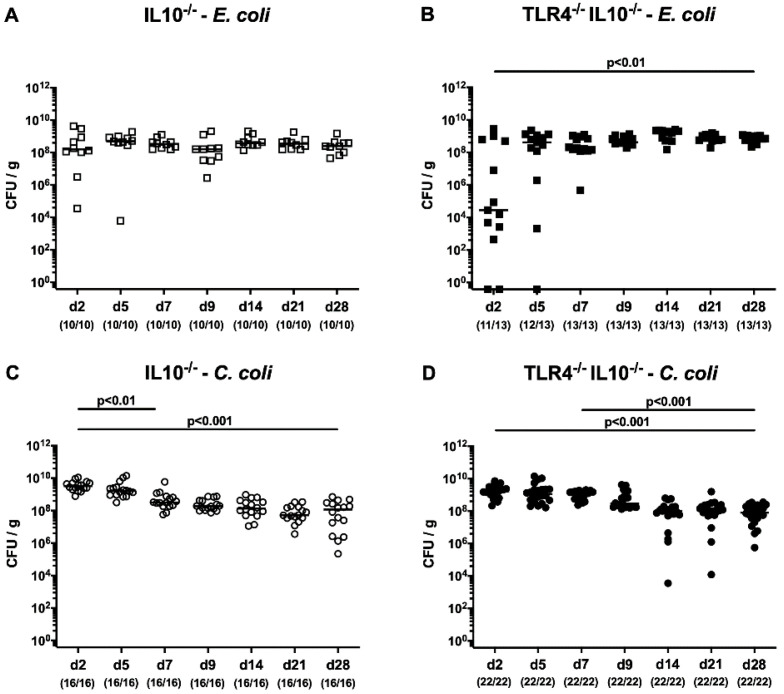
Kinetic survey of fecal loads following peroral *E. coli* or *C. coli* application to secondary abiotic IL-10^−/−^ mice lacking TLR4. Secondary abiotic IL-10^−/−^ mice ((**A**,**C**) open symbols) and IL-10^−/−^ mice lacking TLR4 ((**B**,**D**) TLR4^−/−^ IL-10^−/−^; closed symbols) were perorally challenged with either a commensal murine *E. coli* strain ((**A**,**B**) squares) or a *C. coli* patient isolate ((**C**,**D**) circles) on the day (d) 0 and d1. Intestinal colonization properties were quantitatively surveyed over time in fecal samples taken post-infection by culture (in colony-forming units per g; CFU/g). Medians (black bars), levels of significance (*p*-values) as determined by the Kruskal–Wallis test and Dunn’s post-correction, and numbers of culture-positive mice out of the total number of analyzed animals (in parentheses) are given. Data were pooled from three independent experiments. IL-10, interleukin-10; TLR4, Toll-like receptor-4.

We first surveyed the intestinal colonization properties of pathogenic *C. coli* and commensal *E. coli* following oral challenge of secondary abiotic IL-10^−/−^ mice with or without TLR4. Our cultural analyses revealed stable intestinal colonization efficiencies of either strain that was TLR4-independent, as indicated by high median fecal loads of more than 10^8^ viable bacterial cells per g feces in both IL-10^−/−^ mice lacking TLR4 and IL-10^−/−^ counterparts (Figure 1). From day 2 until day 28 post-challenge, fecal *C. coli* numbers marginally decreased by approximately 1.0–1.5 orders in mice of either genotype (*p* < 0.01–0.001; Figure 1C,D).

**Figure 2:**
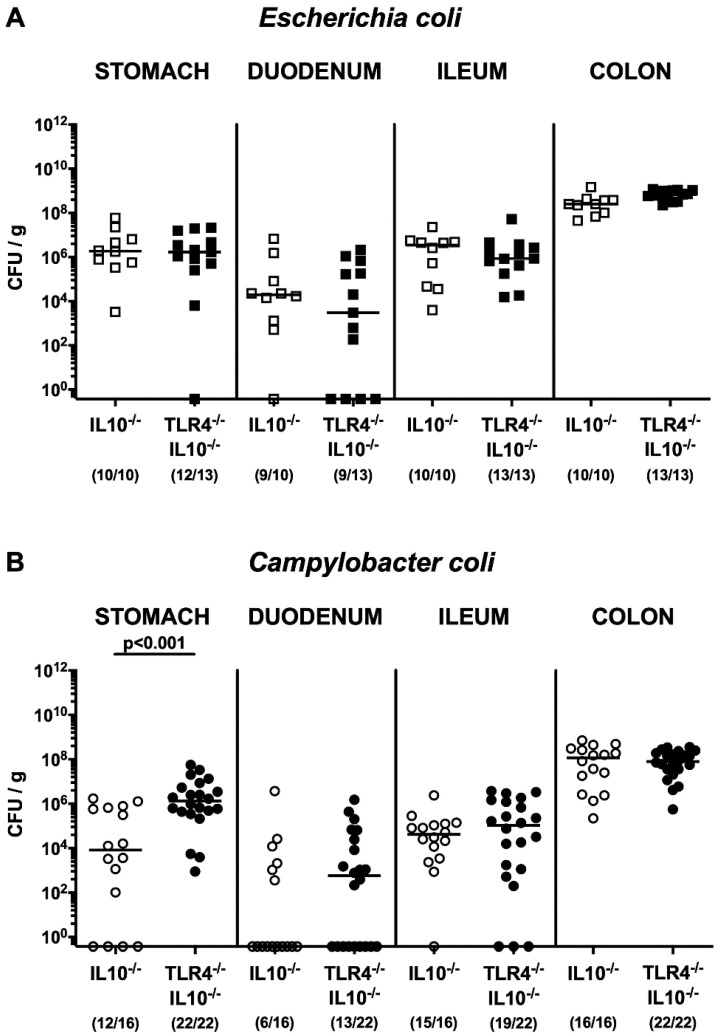
Gastrointestinal *C. coli* and *E. coli* loads following peroral *E. coli* or *C. coli* application to secondary abiotic IL-10^−/−^ mice lacking TLR4. Secondary abiotic IL-10^−/−^ mice (open symbols) and IL-10^−/−^ mice lacking TLR4 (TLR4^−/−^ IL-10^−/−^; closed symbols) were perorally challenged with either a commensal murine *E. coli* strain ((**A**) squares) or a *C. coli* patient isolate ((**B**) circles) on days 0 and 1. On day 28 post-challenge, luminal bacterial loads were quantitatively surveyed in distinct gastrointestinal compartments by culture (in colony-forming units per g; CFU/g). Medians (black bars), levels of significance (*p*-values) as determined by the Mann–Whitney U test, and numbers of culture-positive mice out of the total number of analyzed animals (in parentheses) are given. Data were pooled from three independent experiments.

Upon necropsy, comparable *E. coli* numbers could be cultivated from luminal samples taken from stomach, duodenum, ileum, and colon of TLR4^−/−^ IL-10^−/−^ as compared to IL-10^−/−^ mice (not significant (n.s.); Figure 2A), which also held true for *C. coli*, except for gastric luminal *C. coli* loads that were approximately two orders of magnitude higher in the former as compared to the latter at day 28 post-challenge (*p* < 0.001; Figure 2B). Furthermore, no viable *C. coli* or *E. coli* could be isolated from extra-intestinal tissue sites, such as liver, kidneys, lungs, and cardiac blood (data not shown). Hence, upon peroral application, *C. coli* and *E. coli* stably colonized the murine gastrointestinal tract at high loads in a TLR4-independent fashion.

### 3.2. Kinetic Survey of Clinical Signs Following Peroral C. coli or E. coli Application to Secondary Abiotic IL-10-/- Mice Lacking TLR4

**Figure 3:**
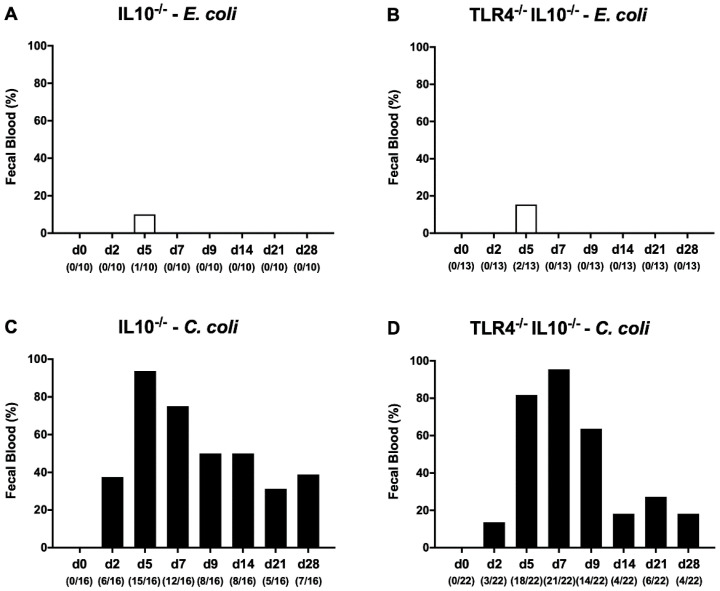
Kinetic survey of fecal blood following peroral *E. coli* or *C. coli* application to secondary abiotic IL-10^−/−^ mice lacking TLR4. Secondary abiotic IL-10^−/−^ mice (**A**,**C**) and IL-10^−/−^ mice lacking TLR4 ((**B**,**D**) TLR4^−/−^ IL-10^−/−^) were perorally challenged with either a commensal murine *E. coli* strain ((**A**,**B**) white bars) or a *C. coli* patient isolate ((**C**,**D**) black bars) on the day (d) 0 and d1. Macroscopic or microscopic detection of fecal blood was surveyed in each mouse over time post-challenge. Bars indicate the frequencies of fecal blood (in%). Numbers of fecal blood-positive mice out of the total number of analyzed animals are given in parentheses. Data were pooled from three independent experiments.

We further quantitatively surveyed clinical signs following peroral bacterial challenge over time by applying a standardized scoring system assessing key features of severe human campylobacteriosis, such as wasting and bloody diarrhea. Whereas mice of either genotype were virtually uncompromised following *E. coli* challenge (Supplementary Figure S1A,B), clinical scores were significantly higher in IL-10^−/−^ mice at days 7 and 28 post-*C. coli* challenge as compared to unchallenged conditions (*p* < 0.001 and *p* < 0.05, respectively), which also held true for day 7 in TLR4^−/−^ IL-10^−/−^ mice (*p* < 0.001; Supplementary Figure S1C,D). Notably, none of the *C. coli*-infected TLR4^−/−^ IL-10^−/−^ mice and only 18.8% of the IL-10^−/−^ counterparts were suffering from acute campylobacteriosis (as indicated by clinical scores of at ≥10), whereas 81.8% and 56.3% of infected TLR4^−/−^ IL-10^−/−^ and IL-10^−/−^ mice remained clinically unaffected, respectively (Supplementary Figure S1C,D). When focusing on the abundance of fecal blood, however (Figure 3), a maximum of 93.8% of IL-10^−/−^ mice were fecal blood-positive as early as 5 days post-challenge (Figure 3C), which was the case for 95.5% of TLR4^−/−^ IL-10^−/−^ mice (Figure 3D), whereas fecal blood was less frequently detected at day 14 and thereafter in the latter as compared to the former (Figure 3C,D). At the end of the observation period, the fecal blood positivity rates were lower in IL-10^−/−^ mice lacking TLR4 as compared to IL-10^−/−^ counterparts (18.2% versus 43.8%; Figure 3C,D). Hence, pathogenic *C. coli* but not commensal *E. coli* caused differential kinetics in fecal bleeding in IL-10^−/−^ and TLR4^−/−^ IL-10^−/−^ mice, indicating that TLR4 was involved in mediating the pathogen-induced signs of intestinal disease.

### 3.3. Role of TLR4 in C. coli-Mediated Colonic Histopathology and Apoptosis

**Figure 4:**
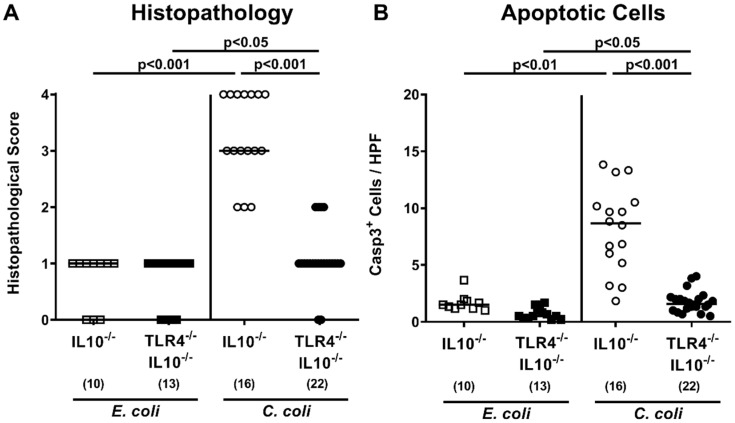
Colonic histopathological and epithelial apoptotic cell responses following peroral *E. coli* or *C. coli* application to secondary abiotic IL-10^−/−^ mice lacking TLR4. Secondary abiotic IL-10^−/−^ mice (open symbols) and IL-10^−/−^ mice lacking TLR4 (TLR4^−/−^ IL-10^−/−^; closed symbols) were perorally challenged with either a commensal murine *E. coli* strain (squares) or a *C. coli* patient isolate (circles) on days 0 and 1. On day 28 post-challenge, (**A**) histopathological changes were quantified in hematoxylin and eosin-stained colonic paraffin sections by applying a standardized scoring system, as described in methods. Furthermore, the average numbers of (**B**) colonic epithelial apoptotic (Casp3^+^) cells were microscopically surveyed in six high power fields (HPF, 400× magnification) per animal in immunohistochemically-stained large intestinal paraffin sections. Medians (black bars), levels of significance (*p*-values) as determined by the Kruskal–Wallis test and Dunn’s post-correction, and numbers of analyzed animals (in parentheses) are indicated. Data were pooled from three independent experiments.

We next assessed potential TLR4-dependent inflammatory responses affecting the large intestines upon *C. coli* versus *E. coli* challenge of secondary abiotic IL-10^−/−^ mice. Therefore, the histopathological changes within the colon were quantified by applying an established histopathological scoring system [41]. On day 28 following *C. coli* infection, higher histopathological scores were assessed in IL-10^−/−^ when compared to TLR4^−/−^ IL-10^−/−^ mice (*p* < 0.001) and also when compared to *E. coli* challenged IL-10^−/−^ control mice (*p* < 0.001; Figure 4A; Supplementary Figure S2A).

Since apoptosis is a hallmark of *C. jejuni*-induced murine campylobacteriosis [16], we furthermore enumerated apoptotic colonic epithelial cells following in situ immunohistochemistry upon necropsy. In line with the obtained histopathological results, a multifold higher number of caspase3^+^ apoptotic cells were found in colonic epithelia at day 28 following *C. coli* versus *E. coli* challenge of IL-10^−/−^ mice (*p* < 0.05–0.01; Figure 4B; Supplementary Figure S2B). These increases were, however, far less pronounced in IL-10^−/−^ mice lacking TLR4 as compared to IL-10^−/−^ controls (*p* < 0.001; Figure 4B; Supplementary Figure S2B). Hence, TLR4 signaling was involved in mediating *C. coli*-induced colonic histopathological and epithelial cell apoptosis upon peroral infection of secondary abiotic IL-10^−/−^ mice.

### 3.4. TLR4-Dependent Intestinal Immune Cell Responses Induced by C. coli Infection

**Figure 5:**
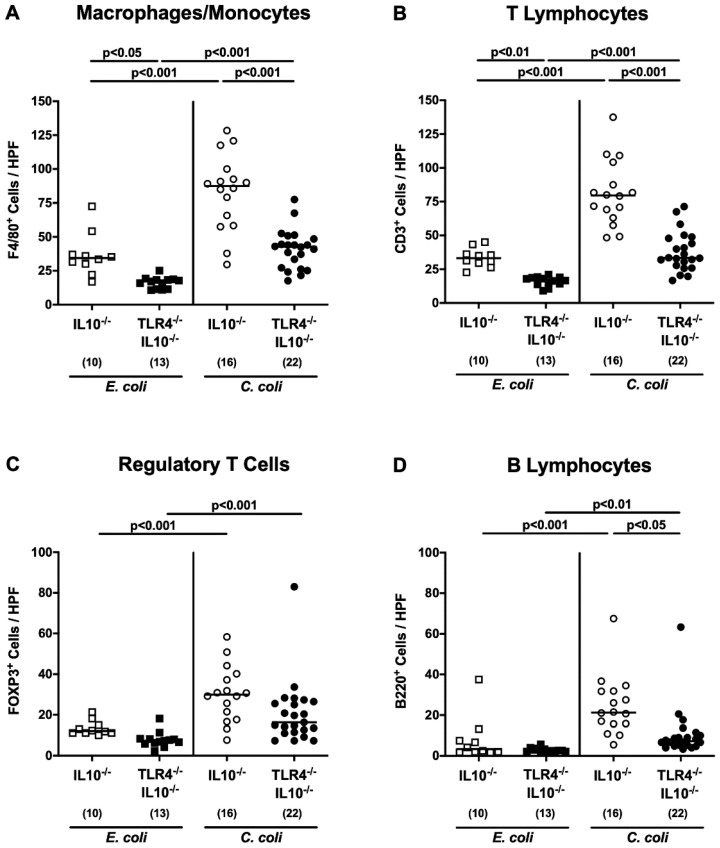
Colonic immune cell responses following peroral *E. coli* or *C. coli* application to secondary abiotic IL-10^−/−^ mice lacking TLR4. Secondary abiotic IL-10^−/−^ mice (open symbols) and IL-10^−/−^ mice lacking TLR4 (TLR4^−/−^ IL-10^−/−^; closed symbols) were perorally challenged with either a commensal murine *E. coli* strain (squares) or a *C. coli* patient isolate (circles) on days 0 and 1. On day 28 post-challenge, the average numbers of (**A**) macrophages and monocytes (F4/80^+^), (**B**) T lymphocytes (CD3^+^), (**C**) regulatory T cells (FOXP3^+^), and (**D**) B lymphocytes (B220^+^) were microscopically surveyed from six high power fields (HPF, 400× magnification) per animal in immunohistochemically-stained colonic paraffin sections. Medians (black bars), levels of significance (*p*-values) as determined by (**A**,**B**) the one-way ANOVA and Tukey’s post-correction and (**C**,**D**) the Kruskal–Wallis test and Dunn’s post-correction, and the numbers of analyzed animals (in parentheses) are indicated. Data were pooled from three independent experiments.

We next surveyed TLR4-dependent innate and adaptive immune responses in the large intestines upon the bacterial challenge of secondary abiotic IL-10^−/−^ mice by applying quantitative in situ immunohistochemistry. On day 28, the numbers of innate immune cell populations, such as F4/80^+^ cells macrophages and monocytes, as well as adaptive immune cell subsets, including CD3^+^ T lymphocytes, FOXP3^+^ regulatory T cells, and B220^+^ B lymphocytes, were higher in the colonic mucosa and lamina propria of *C. coli* as compared to *E. coli* challenged mice of either genotype (*p* < 0.01–0.001; Figure 5; Supplementary Figure S2C–F). Upon either bacterial application, colonic numbers of macrophages and monocytes and of T lymphocytes were lower in TLR4^−/−^ IL-10^−/−^ as compared to respective IL-10^−/−^ counterparts (*p* < 0.01–0.001; Figure 5A,B; Supplementary Figure S2C,D), which also held true for large intestinal B lymphocyte counts at day 28 following *C. coli* infection (*p* < 0.05; Figure 5D; Supplementary Figure S2F). Hence, *C. coli* induced colonic immune cell responses in a TLR4-dependent manner.

### 3.5. TLR4 is Involved in Mediating Intestinal and Extra-Intestinal Pro-Inflammatory Responses Following C. coli Infection of Secondary Abiotic IL-10^−/−^ Mice

**Figure 6:**
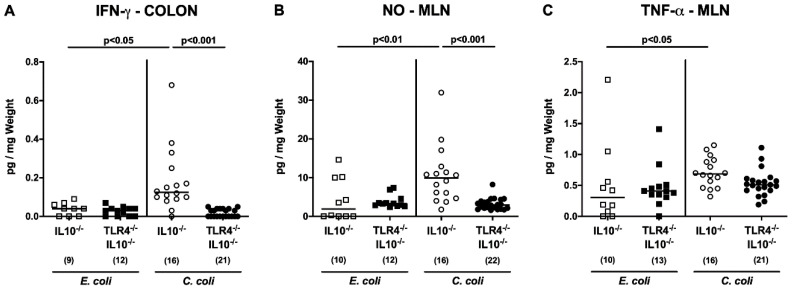
Intestinal pro-inflammatory mediator responses following peroral *E. coli* or *C. coli* application to secondary abiotic IL-10^−/−^ mice lacking TLR4. Secondary abiotic IL-10^−/−^ mice (open symbols) and IL-10^−/−^ mice lacking TLR4 (TLR4^−/−^ IL-10^−/−^; closed symbols) were perorally challenged with either a commensal murine *E. coli* strain (squares) or a *C. coli* patient isolate (circles) on days 0 and 1. On day 28 post-challenge, pro-inflammatory mediators, such as (**A**) IFN-γ, (**B**) nitric oxide (NO), and (**C**) TNF-α were determined in ex vivo biopsies taken from the colon (**A**) and mesenteric lymph nodes (MLN; (**B**,**C**)). Medians (black bars), levels of significance (*p*-values) as determined by the Kruskal–Wallis test and Dunn’s post-correction, and the numbers of analyzed animals (in parentheses) are indicated. Definite outliers were removed after identification with Grubb’s test (α = 0.001). Data were pooled from three independent experiments. IFN-γ, interferon-gamma; TNF, tumor necrosis factor.

We next addressed whether intestinal pro-inflammatory mediator secretion upon bacterial application to IL-10^−/−^ mice also occurred TLR4 dependently. In *C. coli*, as compared to *E. coli*, challenged IL-10^−/−^ but not TLR4-deficient IL-10^−/−^ mice, higher colonic IFN-γ concentrations could be measured (*p* < 0.05; Figure 6A), which also held true for NO and TNF-α secretion in MLN at day 28 post-challenge (*p* < 0.01 and *p* < 0.05, respectively; Figure 6B,C). Moreover, IFN-γ and NO concentrations were lower in ex vivo biopsies taken from colon and MLN, respectively, of *C. coli*-infected TLR4^−/−^ IL-10^−/−^ as compared to IL-10^−/−^ control mice (*p* < 0.001; Figure 6A,B).

**Figure 7:**
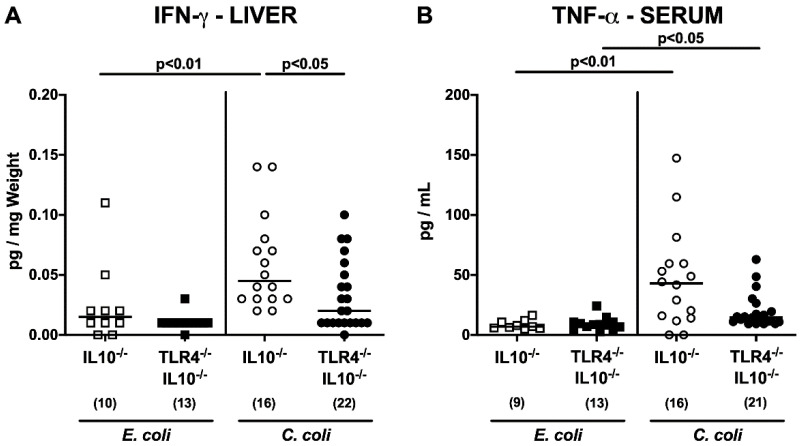
Extra-intestinal including systemic pro-inflammatory cytokine responses following peroral *E. coli* or *C. coli* application to secondary abiotic IL-10^−/−^ mice lacking TLR4. Secondary abiotic IL-10^−/−^ mice (open symbols) and IL-10^−/−^ mice lacking TLR4 (TLR4^−/−^ IL-10^−/−^; closed symbols) were perorally challenged with either a commensal murine *E. coli* strain (squares) or a *C. coli* patient isolate (circles) on days 0 and 1. On day 28 post-challenge, (**A**) hepatic IFN-γ and (**B**) serum TNF-α concentrations were measured. Medians (black bars), levels of significance (*p*-values) as determined by the Kruskal–Wallis test and Dunn’s post-correction, and numbers of analyzed animals (in parentheses) are indicated. Definite outliers were removed after identification with Grubb’s test (α = 0.001). Data were pooled from three independent experiments.

We further asked whether TLR4-dependent pro-inflammatory mediator responses were restricted to the intestinal tract or could also be observed in extra-intestinal, including systemic tissue sites. IFN-γ concentrations in livers were higher in 28 days following *C. coli* as compared to *E. coli* application to IL-10^−/−^ mice (*p* < 0.01; Figure 7A), whereas lower hepatic IFN-γ levels could be obtained from *C. coli*-infected TLR4^−/−^ IL-10^−/−^ versus IL-10^−/−^ control mice (*p* < 0.05; Figure 7A). In serum samples taken from both *C. coli*-infected IL-10^−/−^ mice lacking TLR4 and IL-10^−/−^ counterparts, lower TNF-α concentrations were measured as compared to the respective *E. coli* cohorts (*p* < 0.01–0.05; Figure 7B). Furthermore, a trend towards lower serum TNF-α levels could be observed at day 28 following *C. coli* infection of TLR4^−/−^ IL-10^−/−^ as compared to IL-10^−/−^ mice (n.s.; Figure 7B). Hence, upon *C. coli* infection of secondary abiotic IL-10^−/−^ mice, TLR4-dependent pro-inflammatory mediator responses could be observed in intestinal and extra-intestinal tissue sites, including the liver.

## 4. Discussion

*C. coli* constitutes the second most prevalent causative agent of human *Campylobacter* infections after *C. jejuni* and was recently reported to account for approximately 10% of the confirmed campylobacteriosis cases in the European Union [8]. Yet, little is known about the crosstalk between *C. coli* and the immune system of the vertebrate host, and the lack of molecular data concerning bacterial factors triggering campylobacteriosis underlines the urgent need for a convenient murine model of *C. coli* infection. In the present study, we, therefore, analyzed the pathogenic potential of *C. coli* in the secondary abiotic IL-10^−/−^ murine infection model, which was successfully established and further optimized as one of the valid clinical murine models for acute *C. jejuni* infection, mimicking key features symptoms of severe campylobacteriosis in humans. This acute infection and inflammation model has unraveled the major role of LOS in *C. jejuni*-induced immunopathologies and is nowadays used for the evaluation and validation of novel therapeutic intervention strategies against campylobacteriosis, as shown in recent studies [46,47,48,49]. Whereas several in vitro investigations have proven that *C. jejuni* LOS activates TLR4 in different avian, murine, and human cell lines [19,50,51], only one study to the best of our knowledge revealed that a *C. coli* chicken isolate could activate TLR4 in vitro [20].

In the present study, we provided evidence for TLR4-dependent *C. coli* vertebrate-host interactions in vivo and determined the pathogenic potential of *C. coli* by comparison of data obtained from colonization of mice with an apathogenic commensal *E. coli* isolate. Results indicated that both *C. coli* and *E. coli* stably colonized the small and large intestines of IL-10^−/−^ mice with high loads up to four weeks post-challenge in a TLR4-independent manner. Stable bacterial establishment within the intestinal lumen was accompanied by an abundance of fecal blood in mice of either genotype that had been challenged with *C. coli* as opposed to *E. coli*, which was most prominent within the first week. The finding that *C. coli* but not *E. coli* induced signs of intestinal inflammation proved the pathogenic potential of *C. coli*, which was mediated by the barrier-breaking properties, such as motility, adhesion, and invasion, as demonstrated earlier for *C. jejuni* in the same murine model of infection [52,53]. In TLR4-deficient IL-10^−/−^ mice, however, the maximum fecal blood positivity rates occurred two days later than in infected IL-10^−/−^ counterparts (day 5 versus day 7 p.i.), whereas in the later stage, i.e., from week two to four of *C. coli* infection, fecal blood could less frequently be detected in the former versus the latter. The role of TLR4 in *C. coli*-induced disease outcome is supported by less distinct histopathological changes in the large intestinal tract and by lower numbers of apoptotic epithelial cells in the colon of TLR4^−/−^ IL-10^−/−^ mice versus IL-10^−/−^ mice at day 28 post-*C. coli* challenge. Given that TLR4 can potently induce cell apoptosis [16,17,54], our finding provided evidence that *C. coli* LOS was involved in triggering the inflammatory scenario in the intestines upon infection.

The role of LOS as a virulence factor was further confirmed by the findings that *C. coli* induced both innate and adaptive immune cell responses in a TLR4-dependent manner. Compared to the *E. coli* controls, *C. coli*-infected IL-10^−/−^ mice displayed elevated levels of innate immune cells, such as macrophages and monocytes, which also held true for adaptive immune cells populations, including T and B lymphocytes, which were lower in the TLR4^−/−^ IL-10^−/−^ cohort at day 28 p.i. These results are supported by our previous studies showing TLR4-dependent immune cell responses upon infection with *C. jejuni* 81-176 in the same murine model of infection [17], as well as in secondary abiotic wildtype mice [16,17]. This confirms that *C. coli* induces campylobacteriosis similar to *C. jejuni* in a TLR4-dependent manner and that LOS plays a major role in *C. coli* immunopathogenesis.

Furthermore, in line with results derived from *C. jejuni*-infected IL-10^−/−^ mice suffering from acute enterocolitis within the first week of infection [17], pro-inflammatory mediator secretion was less pronounced in intestinal ex vivo biopsies derived from TLR4-deficient IL-10^−/−^ mice as compared to IL-10^−/−^ controls at day 28 post-*C. coli* infection. Remarkably, TLR4-dependent immune responses were not restricted to the intestinal tract but could also be observed in extra-intestinal compartments, as indicated by less hepatic IFN-γ secretion in *C. coli*-infected TLR4-deficient IL-10^−/−^ mice as compared to IL-10^−/−^ mice. Interestingly, no viable *C. coli* that might have translocated from the lumen of the inflamed intestines could be isolated from extra-intestinal tissue sites including the blood stream at all. We could not exclude, however, that translocated bacteria would most likely have been cleared by the immune system as late as day 28 p.i. One needs to take further into consideration that soluble *C. coli* constituents, including LOS, might exert potent pro-inflammatory effects at any extra-intestinal, including systemic tissue sites [55].

The here presented results are well in line with our very recent study, where we addressed the role of TLR4 in *C. coli*-infected mice harboring a complex human gut microbiota [36]. The pathogen could establish within the gastrointestinal tract of both IL-10^−/−^ and TLR4-deficient IL-10^−/−^ mice until day 21 p.i., but induced less pronounced immunopathological sequelae in the latter versus the former upon oral challenge. This study provided the first evidence for the pivotal role of TLR4-dependent *C. coli*-host responses in concert with the human gut microbiota [36]. Our actual study involving microbiota-depleted mice, however, further underlined the important immunopathological role of TLR4 in mediating pathogenic LOS responses in the absence of any other TLR4 ligands derived from the commensal gut microbiota.

The auxiliary finding that TLR4-deficient IL-10^−/−^ mice harbored approximately two orders of magnitude higher *C. coli* bacterial numbers in the stomach as compared to their IL-10^−/−^ counterparts provides evidence that TLR4 triggers immune defense mechanisms in the stomach to protect from pathogens and to maintain intestinal homeostasis [56]. The basolateral expression has been shown for several TLRs, resulting in less frequent activation by stimuli derived from luminal bacteria [57]. One study revealed that TLR4 is mainly localized at the basolateral surface of the colonic epithelium in a healthy murine intestine, whereas another study showed that TLR4 is expressed both on the apical and basolateral surface of the gastric epithelium in mice infected with *Helicobacter pylori*, which is closely related to *Campylobacter* [58,59]. In this scenario, the basolateral expression of TLR4 could possibly allow *C. coli* to colonize at higher levels. However, it cannot be excluded that apical TLR4 expression is induced upon an increase in LPS or LOS from Gram-negative pathogens.

In summary, our results demonstrate that TLR4 signaling is involved in mediating the inflammatory responses upon *C. coli* infection in the vertebrate host. However, we cannot exclude that also other TLRs are participating in the recognition of this pathogen, such as TLR2, TLR5, and TLR9, recognizing bacterial lipoprotein, flagella, and CpG-DNA, respectively [60]. The *C. coli* chicken isolate investigated by Zoete et al. could activate TLR2 and additionally TLR21, which is the receptor recognizing CpG-DNA in poultry but not TLR5 and TLR9 [20]. Moreover, our group showed earlier that besides TLR4, also TLR2 and TLR9 play a role in mediating *C. jejuni*-induced immunopathology [16,17]. In addition, secondary abiotic IL-10^−/−^ mice deficient in the innate receptor nucleotide-oligonucleotide-domain 2 (Nod2) developed less severe enterocolitis upon peroral *C. jejuni* infection [35]. Although *C. jejuni* is able to evade recognition by TLR5 [61], we do not know whether this also may apply to *C. coli*. Furthermore, in order to unravel potential strain-specific host interactions, it is necessary to include *C. coli* strains isolated from different sources. One study, for instance, assessed the colonization abilities of several *C. coli* strains isolated from three different sources, namely symptomatic carriers, asymptomatic carriers, and chicken carcasses, and revealed marked functional differences between the strains, which were most likely source-dependent [62]. It would, therefore, be of high interest to unravel whether these differences in colonization capacities can be attributed to LOS diversity, affecting host TLR4 recognition.

## 5. Conclusions

We further conclude that the here applied infection model of LOS-sensitized secondary abiotic IL-10^−/−^ mice, which had initially been established to unravel *C. jejuni*-host interactions, mimicking severe human campylobacteriosis, can also be used to study the molecular mechanism underlying *C. coli*-induced immunopathogenesis and, hence, paves the way to develop therapeutic and prophylactic options to combat campylobacteriosis as well as post-infectious sequelae.

## Supplementary Materials

The following are available online at https://www.mdpi.com/2076-2607/8/12/1882/s1, Figure S1: Kinetic survey of clinical conditions following peroral *E. coli* or *C. coli* application to secondary abiotic IL-10^−/−^ mice lacking TLR4, Figure S2: Representative photomicrographs of H&E and immunohistochemically stained colonic paraffin sections.

## Abbreviations

CBA	cytometric bead array
CFU	colony forming units
HPF	high power fields
IFN	interferon
IL	interleukin
LOS	lipooligosaccharide
LPS	lipopolysaccharide
MCP-1	monocyte chemoattractant protein 1
MLN	mesenteric lymph nodes
NO	nitric oxide
n.s.	not significant
PBS	phosphate-buffered saline
p.i.	post-infection
SPF	specific pathogen free
TLR	Toll-like receptor
TNF	tumor necrosis factor
Treg	regulatory T cells
WT	wildtype

## References

[1] Wagenaar J.A., French N.P., Havelaar A.H. (2013). Preventing Campylobacter at the source: Why is it so difficult?. Clin. Infect. Dis..

[2] World Health Organization. Campylobacter. https://www.who.int/news-room/fact-sheets/detail/campylobacter.

[3] Young K.T., Davis L.M., Dirita V.J. (2007). *Campylobacter jejuni*: *Molecular* biology and pathogenesis. Nat. Rev. Microbiol..

[4] Backert S., Tegtmeyer N., Cróinín T., Boehm M., Heimesaat M. (2017). Human Campylobacteriosis.

[5] Allos B.M. (1997). Association between *Campylobacter* infection and Guillain-Barre syndrome. J. Infect. Dis..

[6] Kist M., Bereswill S. (2001). Campylobacter jejuni. Emerging Bacterial Pathogens.

[7] Silva J., Leite D., Fernandes M., Mena C., Gibbs P.A., Teixeira P. (2011). Campylobacter spp. as a foodborne pathogen: A review. Front. Microbiol..

[8] European Food Safety Authority, European Centre for Disease Prevention and Control (2019). The European Union One Health 2018 Zoonoses Report. EFSA J..

[9] Wilson D.J., Gabriel E., Leatherbarrow A.J.H., Cheesbrough J., Gee S., Bolton E., Fox A., Fearnhead P., Hart C.A., Diggle P.J. (2008). Tracing the source of campylobacteriosis. PLoS Genet..

[10] Alter T., Bereswill S., Glünder G., Haag L.-M., Hänel I., Heimesaat M., Lugert R., Rautenschlein S., Weber R., Zautner A. (2011). Die Campylobacteriose des Menschen. Bundesgesundheitsblatt Gesundh. Gesundh..

[11] Kaakoush N.O., Castaño-Rodríguez N., Mitchell H.M., Man S.M. (2015). Global Epidemiology of Campylobacter Infection. Clin. Microbiol. Rev..

[12] Tam C.C., O’Brien S.J., Adak G.K., Meakins S.M., Frost J.A. (2003). Campylobacter coli—An important foodborne pathogen. J. Infect..

[13] Chaplin D.D. (2010). Overview of the immune response. J. Allergy Clin. Immunol..

[14] Tan Y., Kagan J.C. (2014). A cross-disciplinary perspective on the innate immune responses to bacterial lipopolysaccharide. Mol. Cell.

[15] Ramachandran G. (2014). Gram-positive and gram-negative bacterial toxins in sepsis: A brief review. Virulence.

[16] Bereswill S., Fischer A., Plickert R., Haag L.M., Otto B., Kuhl A.A., Dasti J.I., Zautner A.E., Munoz M., Loddenkemper C. (2011). Novel murine infection models provide deep insights into the “menage a trois” of *Campylobacter jejuni*, microbiota and host innate immunity. PLoS ONE.

[17] Haag L.M., Fischer A., Otto B., Plickert R., Kuhl A.A., Gobel U.B., Bereswill S., Heimesaat M.M. (2012). *Campylobacter jejuni* induces acute enterocolitis in gnotobiotic IL-10-/- mice via Toll-like-receptor-2 and -4 signaling. PLoS ONE.

[18] Stahl M., Ries J., Vermeulen J., Yang H., Sham H.P., Crowley S.M., Badayeva Y., Turvey S.E., Gaynor E.C., Li X. (2014). A novel mouse model of *Campylobacter jejuni* gastroenteritis reveals key pro-inflammatory and tissue protective roles for Toll-like receptor signaling during infection. PLoS Pathog..

[19] Rathinam V.A., Appledorn D.M., Hoag K.A., Amalfitano A., Mansfield L.S. (2009). *Campylobacter jejuni*-induced activation of dendritic cells involves cooperative signaling through Toll-like receptor 4 (TLR4)-MyD88 and TLR4-TRIF axes. Infect. Immun..

[20] de Zoete M.R., Keestra A.M., Roszczenko P., van Putten J.P. (2010). Activation of human and chicken toll-like receptors by Campylobacter spp.. Infect. Immun..

[21] Mortensen N.P., Kuijf M.L., Ang C.W., Schiellerup P., Krogfelt K.A., Jacobs B.C., van Belkum A., Endtz H.P., Bergman M.P. (2009). Sialylation of *Campylobacter jejuni* lipo-oligosaccharides is associated with severe gastro-enteritis and reactive arthritis. Microbes Infect..

[22] Preston A., Mandrell R.E., Gibson B.W., Apicella M.A. (1996). The lipooligosaccharides of pathogenic gram-negative bacteria. Crit. Rev. Microbiol..

[23] Rietschel E.T., Kirikae T., Schade F.U., Mamat U., Schmidt G., Loppnow H., Ulmer A.J., Zahringer U., Seydel U., Di Padova F. (1994). Bacterial endotoxin: Molecular relationships of structure to activity and function. FASEB J..

[24] Culebro A., Revez J., Pascoe B., Friedmann Y., Hitchings M.D., Stupak J., Sheppard S.K., Li J., Rossi M. (2016). Large sequence diversity within the biosynthesis locus and common biochemical features of *Campylobacter coli* lipooligosaccharides. J Bacteriol.

[25] Richards V.P., Lefebure T., Pavinski Bitar P.D., Stanhope M.J. (2013). Comparative characterization of the virulence gene clusters (lipooligosaccharide [LOS] and capsular polysaccharide [CPS]) for Campylobacter coli, Campylobacter jejuni subsp. jejuni and related Campylobacter species. Infect. Genet. Evol..

[26] Culebro A., Machado M.P., Carriço J.A., Rossi M. (2018). Origin, evolution, and distribution of the molecular machinery for biosynthesis of sialylated lipooligosaccharide structures in Campylobacter coli. Sci. Rep..

[27] Haag A.F., Baloban M., Sani M., Kerscher B., Pierre O., Farkas A., Longhi R., Boncompagni E., Herouart D., Dall’angelo S. (2011). Protection of Sinorhizobium against host cysteine-rich antimicrobial peptides is critical for symbiosis. PLoS Biol..

[28] Haag L.M., Fischer A., Otto B., Grundmann U., Kuhl A.A., Gobel U.B., Bereswill S., Heimesaat M.M. (2012). *Campylobacter jejuni* infection of infant mice: Acute enterocolitis is followed by asymptomatic intestinal and extra-intestinal immune responses. Eur. J. Microbiol. Immunol..

[29] Alutis M.E., Grundmann U., Fischer A., Kuhl A.A., Bereswill S., Heimesaat M.M. (2014). Selective gelatinase inhibition reduces apoptosis and pro-inflammatory immune cell responses in Campylobacter jejuni-infected gnotobiotic IL-10 deficient mice. Eur. J. Microbiol. Immunol..

[30] Bereswill S., Ekmekciu I., Escher U., Fiebiger U., Stingl K., Heimesaat M.M. (2017). *Lactobacillus johnsonii* ameliorates intestinal, extra-intestinal and systemic pro-inflammatory immune responses following murine *Campylobacter jejuni* infection. Sci. Rep..

[31] Bereswill S., Grundmann U., Alutis M.E., Fischer A., Heimesaat M.M. (2017). *Campylobacter jejuni* infection of conventionally colonized mice lacking nucleotide-oligomerization-domain-2. Gut Pathog..

[32] Ekmekciu I., Fiebiger U., Stingl K., Bereswill S., Heimesaat M.M. (2017). Amelioration of intestinal and systemic sequelae of murine Campylobacter jejuni infection by probiotic VSL#3 treatment. Gut Pathog..

[33] Heimesaat M.M., Alutis M., Grundmann U., Fischer A., Tegtmeyer N., Bohm M., Kuhl A.A., Gobel U.B., Backert S., Bereswill S. (2014). The role of serine protease HtrA in acute ulcerative enterocolitis and extra-intestinal immune responses during *Campylobacter jejuni* infection of gnotobiotic IL-10 deficient mice. Front. Cell. Infect. Microbiol..

[34] Heimesaat M.M., Grundmann U., Alutis M.E., Fischer A., Bereswill S. (2017). Small Intestinal Pro-Inflammatory Immune Responses Following *Campylobacter Jejuni* Infection of Secondary Abiotic IL-10^(−/−)^ Mice Lacking Nucleotide-Oligomerization-Domain-2. Eur. J. Microbiol. Immunol..

[35] Heimesaat M.M., Grundmann U., Alutis M.E., Fischer A., Bereswill S. (2017). Absence of Nucleotide-Oligomerization-Domain-2 Is Associated with Less Distinct Disease in Campylobacter jejuni Infected Secondary Abiotic IL-10 Deficient Mice. Front. Cell. Infect. Microbiol..

[36] Kløve S., Genger C., Mousavi S., Weschka D., Bereswill S., Heimesaat M.M. (2020). Toll-Like Receptor-4 Dependent Intestinal and Systemic Sequelae Following Peroral *Campylobacter coli* Infection of IL10 Deficient Mice Harboring a Human Gut Microbiota. Pathogens.

[37] Heimesaat M.M., Bereswill S., Fischer A., Fuchs D., Struck D., Niebergall J., Jahn H.K., Dunay I.R., Moter A., Gescher D.M. (2006). Gram-negative bacteria aggravate murine small intestinal Th1-type immunopathology following oral infection with *Toxoplasma gondii*. J. Immunol..

[38] Heimesaat M.M., Fischer A., Jahn H.K., Niebergall J., Freudenberg M., Blaut M., Liesenfeld O., Schumann R.R., Gobel U.B., Bereswill S. (2007). Exacerbation of murine ileitis by Toll-like receptor 4 mediated sensing of lipopolysaccharide from commensal *Escherichia coli*. Gut.

[39] Haag L.M., Fischer A., Otto B., Plickert R., Kuhl A.A., Gobel U.B., Bereswill S., Heimesaat M.M. (2012). Intestinal microbiota shifts towards elevated commensal Escherichia coli loads abrogate colonization resistance against Campylobacter jejuni in mice. PLoS ONE.

[40] Heimesaat M.M., Haag L.M., Fischer A., Otto B., Kuhl A.A., Gobel U.B., Bereswill S. (2013). Survey of extra-intestinal immune responses in asymptomatic long-term Campylobacter jejuni-infected mice. Eur. J. Microbiol. Immunol..

[41] Erben U., Loddenkemper C., Doerfel K., Spieckermann S., Haller D., Heimesaat M.M., Zeitz M., Siegmund B., Kühl A.A. (2014). A guide to histomorphological evaluation of intestinal inflammation in mouse models. Int. J. Clin. Exp. Pathol..

[42] Alutis M.E., Grundmann U., Fischer A., Hagen U., Kuhl A.A., Gobel U.B., Bereswill S., Heimesaat M.M. (2015). The Role of Gelatinases in *Campylobacter Jejuni* Infection of Gnotobiotic Mice. Eur. J. Microbiol. Immunol..

[43] Alutis M.E., Grundmann U., Hagen U., Fischer A., Kuhl A.A., Gobel U.B., Bereswill S., Heimesaat M.M. (2015). Matrix Metalloproteinase-2 Mediates Intestinal Immunopathogenesis in *Campylobacter Jejuni*-Infected Infant Mice. Eur. J. Microbiol. Immunol..

[44] Heimesaat M.M., Lugert R., Fischer A., Alutis M., Kuhl A.A., Zautner A.E., Tareen A.M., Gobel U.B., Bereswill S. (2014). Impact of *Campylobacter jejuni* cj0268c knockout mutation on intestinal colonization, translocation, and induction of immunopathology in gnotobiotic IL-10 deficient mice. PLoS ONE.

[45] Heimesaat M.M., Nogai A., Bereswill S., Plickert R., Fischer A., Loddenkemper C., Steinhoff U., Tchaptchet S., Thiel E., Freudenberg M.A. (2010). MyD88/TLR9 mediated immunopathology and gut microbiota dynamics in a novel murine model of intestinal graft-versus-host disease. Gut.

[46] Mousavi S., Lobo de Sa F.D., Schulzke J.D., Bucker R., Bereswill S., Heimesaat M.M. (2019). Vitamin D in Acute Campylobacteriosis-Results From an Intervention Study Applying a Clinical Campylobacter jejuni Induced Enterocolitis Model. Front. Immunol..

[47] Mousavi S., Schmidt A.M., Escher U., Kittler S., Kehrenberg C., Thunhorst E., Bereswill S., Heimesaat M.M. (2020). Carvacrol ameliorates acute campylobacteriosis in a clinical murine infection model. Gut Pathog..

[48] Mousavi S., Escher U., Thunhorst E., Kittler S., Kehrenberg C., Bereswill S., Heimesaat M.M. (2020). Vitamin C alleviates acute enterocolitis in *Campylobacter jejuni* infected mice. Sci. Rep..

[49] Lobo de Sa F.D., Butkevych E., Nattramilarasu P.K., Fromm A., Mousavi S., Moos V., Golz J.C., Stingl K., Kittler S., Seinige D. (2019). Curcumin Mitigates Immune-Induced Epithelial Barrier Dysfunction by Campylobacter jejuni. Int. J. Mol. Sci..

[50] Watson R.O., Galan J.E. (2005). Signal transduction in *Campylobacter jejuni*-induced cytokine production. Cell. Microbiol..

[51] van Mourik A., Steeghs L., van Laar J., Meiring H.D., Hamstra H.J., van Putten J.P., Wosten M.M. (2010). Altered linkage of hydroxyacyl chains in lipid A of Campylobacter jejuni reduces TLR4 activation and antimicrobial resistance. J. Biol. Chem..

[52] Schmidt A.M., Escher U., Mousavi S., Boehm M., Backert S., Bereswill S., Heimesaat M.M. (2019). Protease Activity of Campylobacter jejuni HtrA Modulates Distinct Intestinal and Systemic Immune Responses in Infected Secondary Abiotic IL-10 Deficient Mice. Front. Cell. Infect. Microbiol..

[53] Schmidt A.M., Escher U., Mousavi S., Tegtmeyer N., Boehm M., Backert S., Bereswill S., Heimesaat M.M. (2019). Immunopathological properties of the *Campylobacter jejuni* flagellins and the adhesin CadF as assessed in a clinical murine infection model. Gut Pathog..

[54] Haase R., Kirschning C.J., Sing A., Schrottner P., Fukase K., Kusumoto S., Wagner H., Heesemann J., Ruckdeschel K. (2003). A dominant role of Toll-like receptor 4 in the signaling of apoptosis in bacteria-faced macrophages. J. Immunol..

[55] González-Navajas J.M., Fine S., Law J., Datta S.K., Nguyen K.P., Yu M., Corr M., Katakura K., Eckman L., Lee J. (2010). TLR4 signaling in effector CD4+ T cells regulates TCR activation and experimental colitis in mice. J. Clin. Investig..

[56] Yu S., Gao N. (2015). Compartmentalizing intestinal epithelial cell toll-like receptors for immune surveillance. Cell. Mol. Life Sci. CMLS.

[57] Abreu M.T. (2010). Toll-like receptor signalling in the intestinal epithelium: How bacterial recognition shapes intestinal function. Nat. Rev. Immunol..

[58] Ortega-Cava C.F., Ishihara S., Rumi M.A.K., Kawashima K., Ishimura N., Kazumori H., Udagawa J., Kadowaki Y., Kinoshita Y. (2003). Strategic Compartmentalization of Toll-Like Receptor 4 in the Mouse Gut. J. Immunol..

[59] Schmausser B., Andrulis M., Endrich S., Lee S.K., Josenhans C., Müller-Hermelink H.K., Eck M. (2004). Expression and subcellular distribution of toll-like receptors TLR4, TLR5 and TLR9 on the gastric epithelium in Helicobacter pylori infection. Clin. Exp. Immunol..

[60] Pandey S., Kawai T., Akira S. (2014). Microbial sensing by Toll-like receptors and intracellular nucleic acid sensors. Cold Spring Harb. Perspect. Biol..

[61] Andersen-Nissen E., Smith K.D., Strobe K.L., Barrett S.L., Cookson B.T., Logan S.M., Aderem A. (2005). Evasion of Toll-like receptor 5 by flagellated bacteria. Proc. Natl. Acad. Sci. USA.

[62] Ciftci A., Savasan S., Ica T., Diker K.S. (2009). Mouse intestine colonization ability of Campylobacter coli strains. Dtsch. Tierarztl. Wochenschr..

