# Supplementary material for: Toll-Like Receptor-4 Is Involved in Mediating Intestinal and Extra-Intestinal Inflammation in Campylobacter coli-Infected Secondary Abiotic IL-10−/− Mice
Source: Microorganisms. 2020 Nov 27;8(12):1882. doi: 10.3390/microorganisms8121882 (PMC7761268; doi:10.3390/microorganisms8121882)
Supplement: Supplementary file 1 [file microorganisms-08-01882-s001.zip › Supplementary_FigS2_COLON_PICS_TLR4-IL10_Ccoli_Ecoli_050920.pdf]

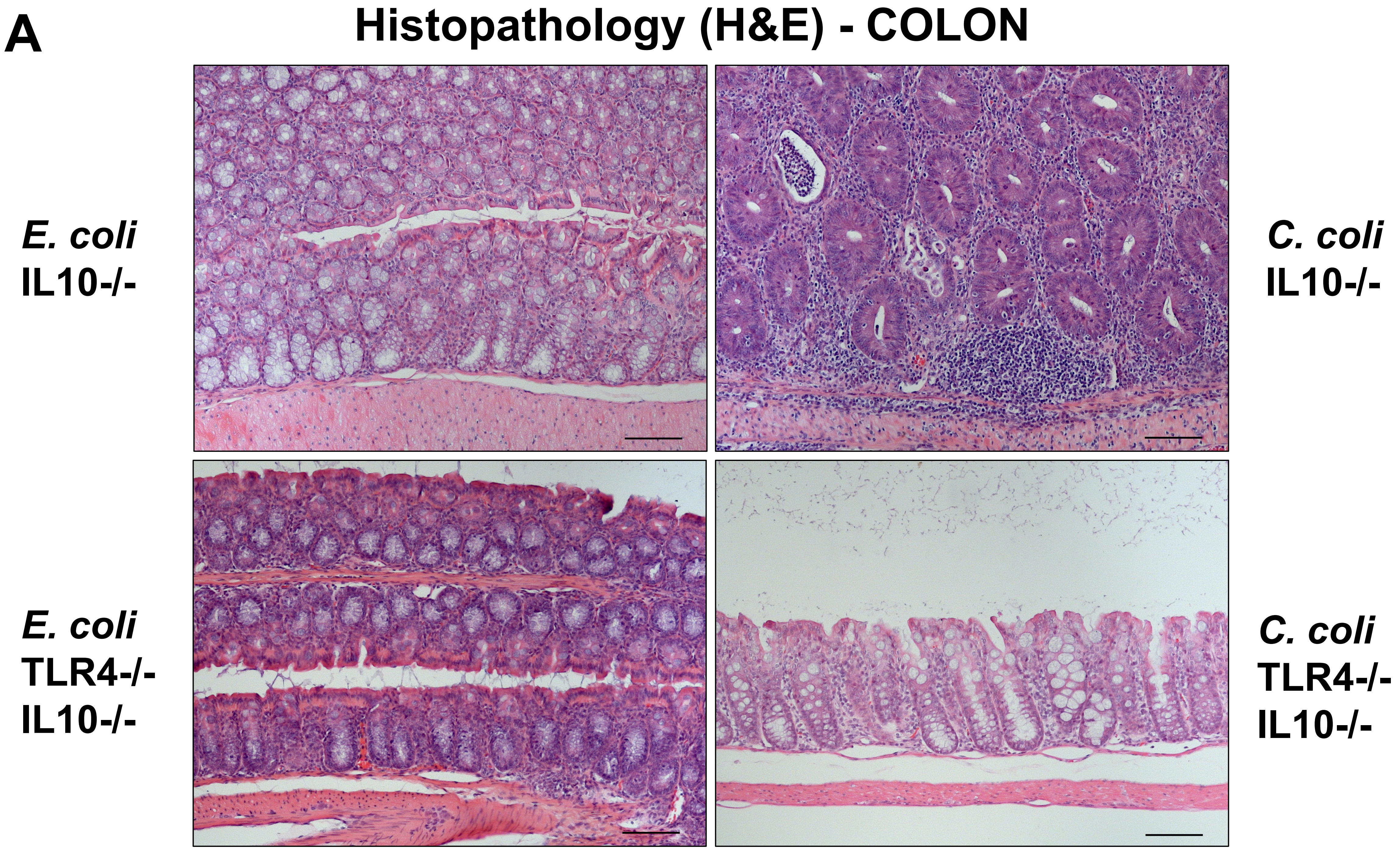
A
Histopathology (H&E) - COLON
E. coli
IL10-/-
C. coli
IL10-/-
E. coli
TLR4-/-
IL10-/-
C. coli
TLR4-/-
IL10-/-

B

Apoptotic Cells (Casp3+) - COLON

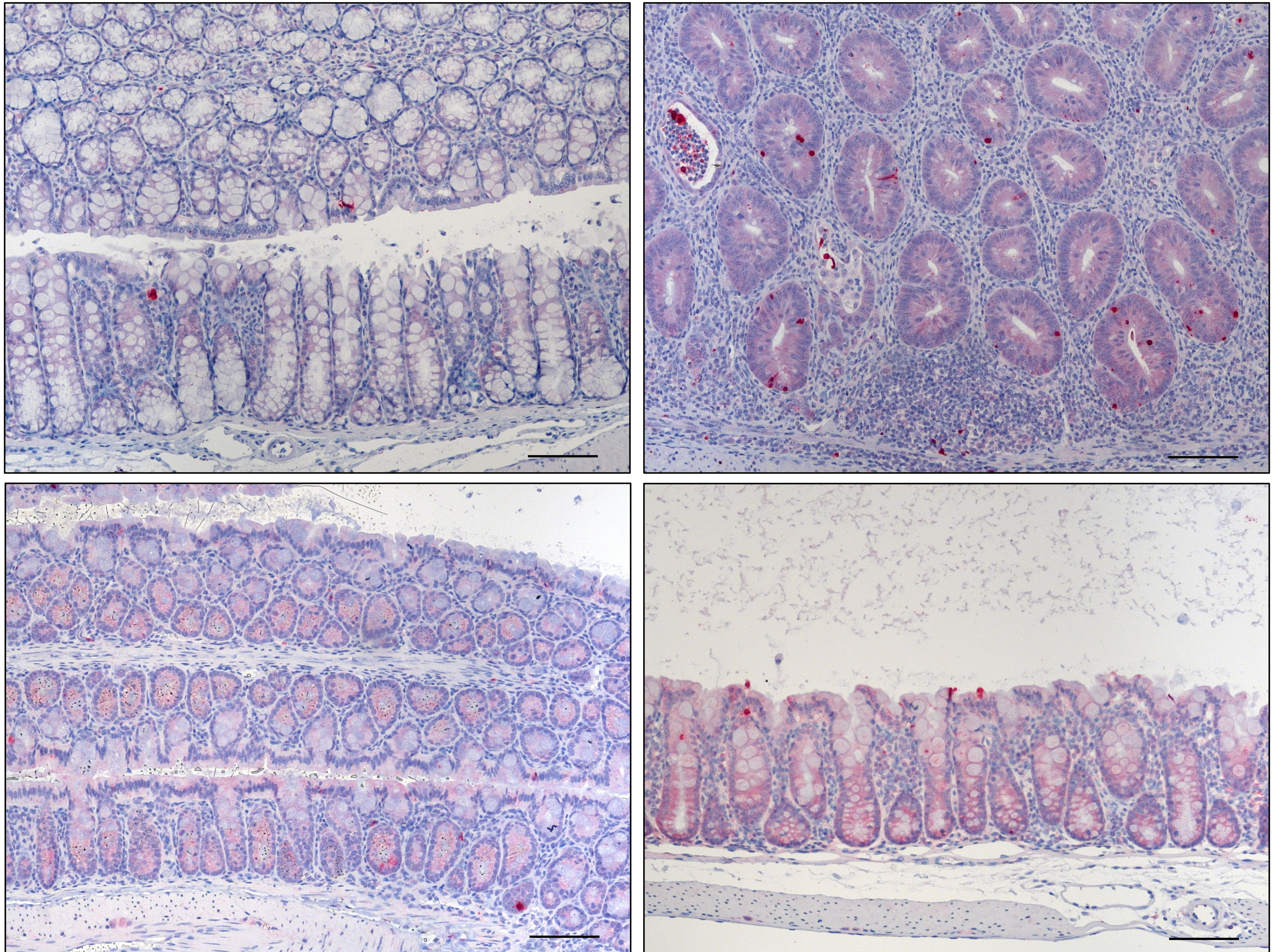

E. coli IL10-/-

C. coli IL10-/-

E. coli TLR4-/- IL10-/-

C. coli TLR4-/- IL10-/-

C

Macrophages / Monocytes (F4/80+) - COLON

E. coli
IL10-/-

C. coli
IL10-/-

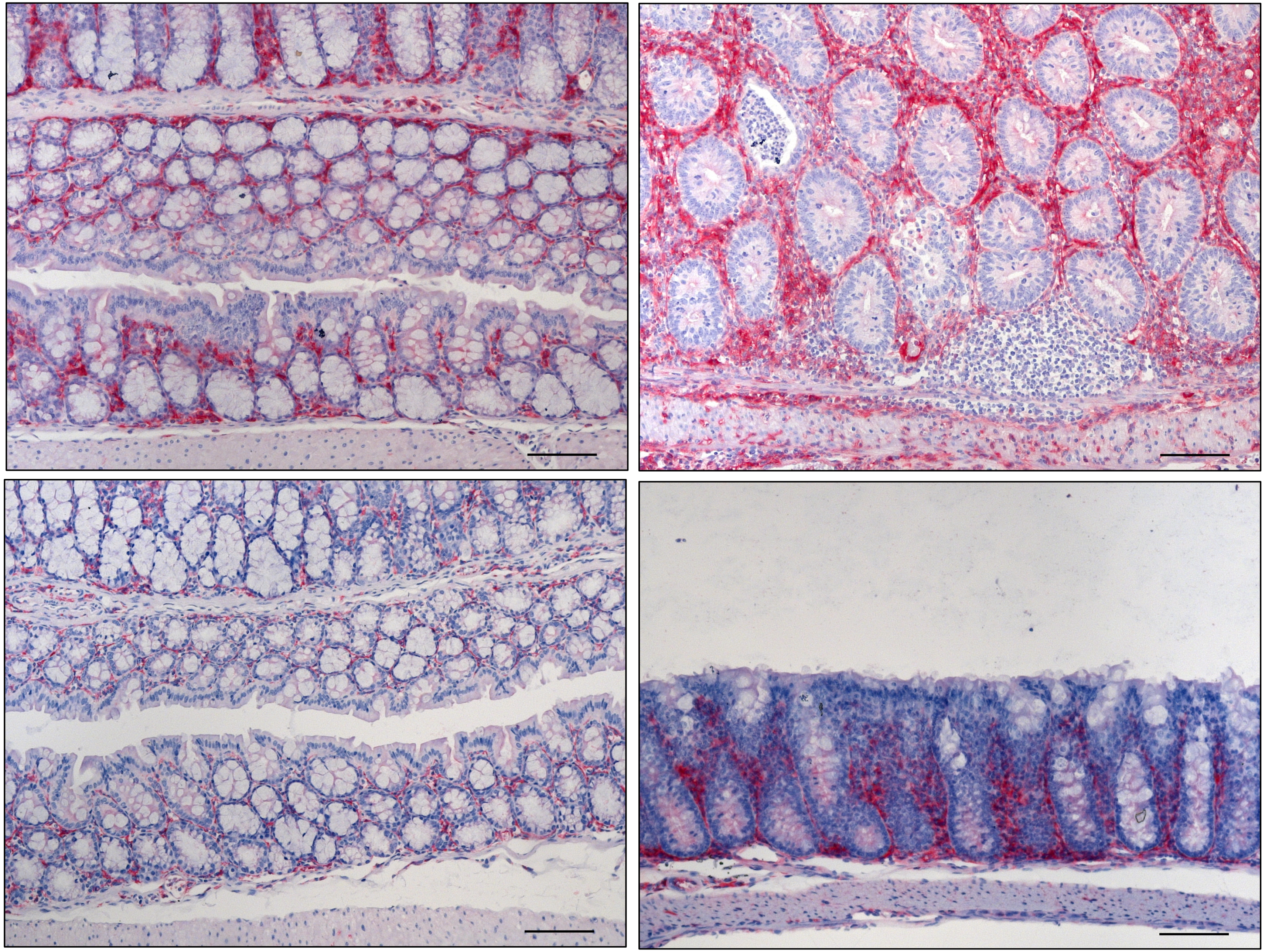

E. coli
TLR4-/-
IL10-/-

C. coli
TLR4-/-
IL10-/-

D

T Lymphocytes (CD3+) - COLON

E. coli **IL10-/-**

C. coli **IL10-/-**

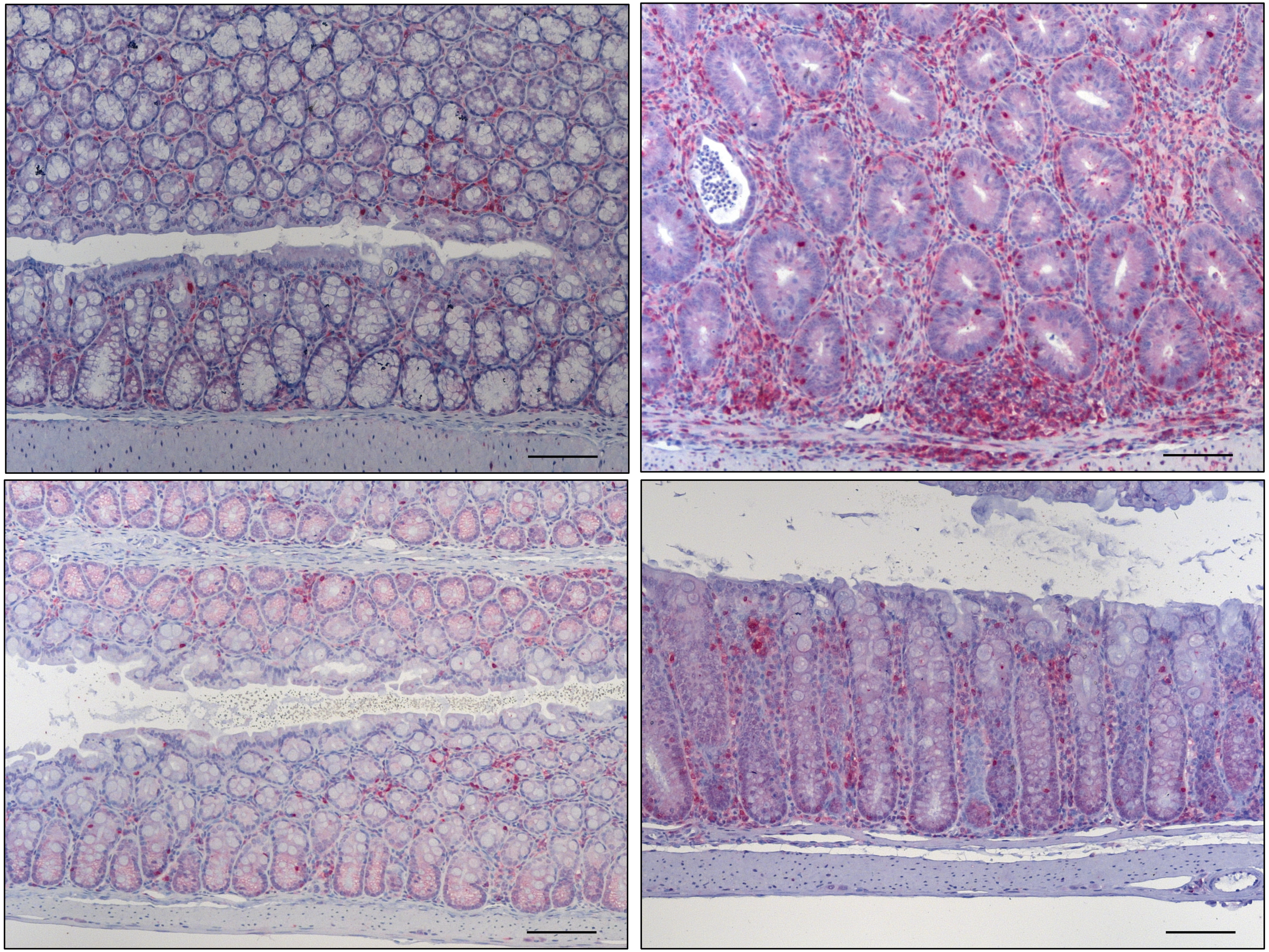

E. coli **TLR4-/- IL10-/-**

C. coli **TLR4-/- IL10-/-**

E

Regulatory T Cells (FOXP3+) - COLON

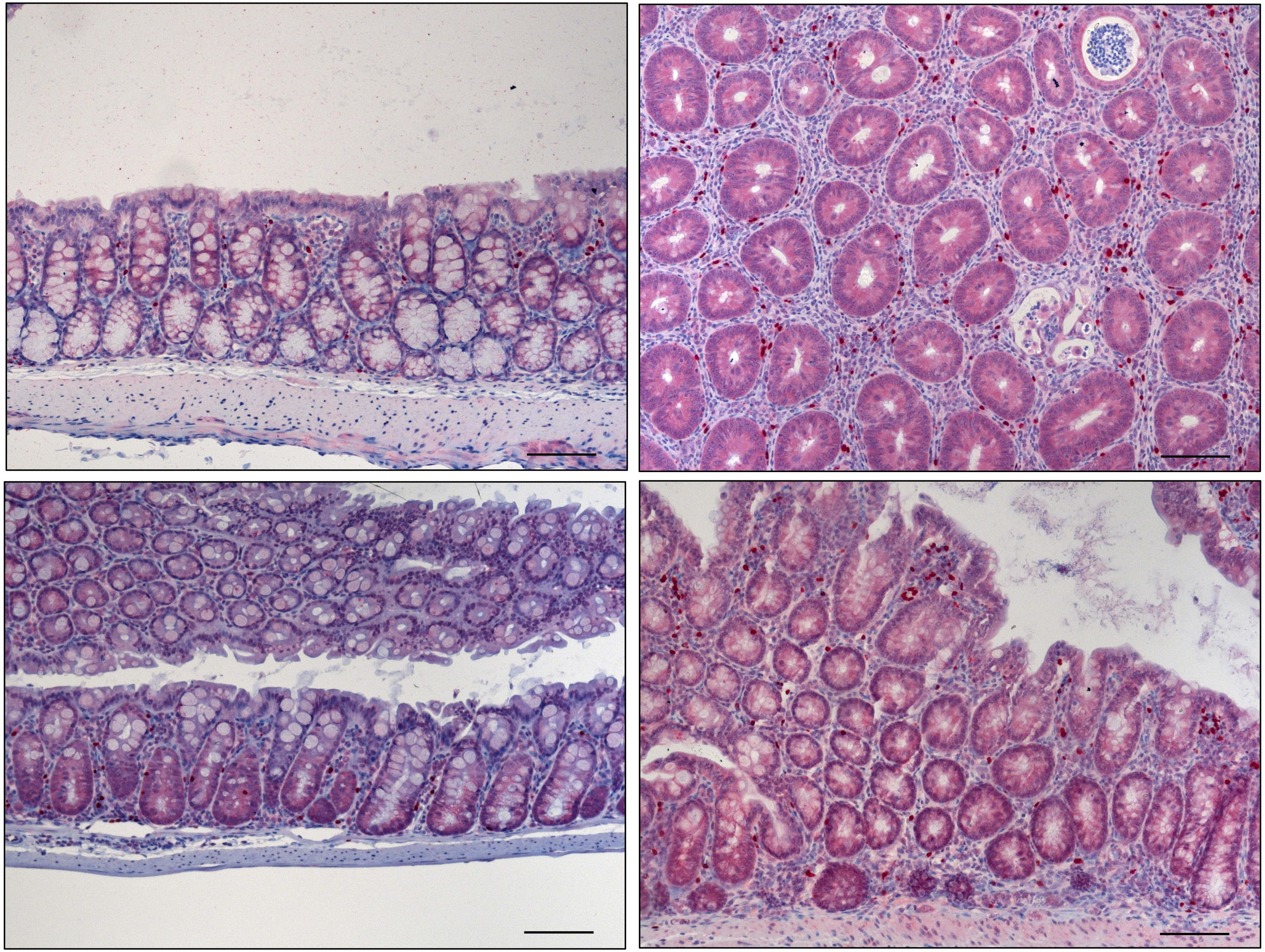

E. coli IL10-/-

C. coli IL10-/-

E. coli TLR4-/- IL10-/-

C. coli TLR4-/- IL10-/-

F

B Lymphocytes (B220+) - COLON

E. coli
IL10-/-

C. coli
IL10-/-

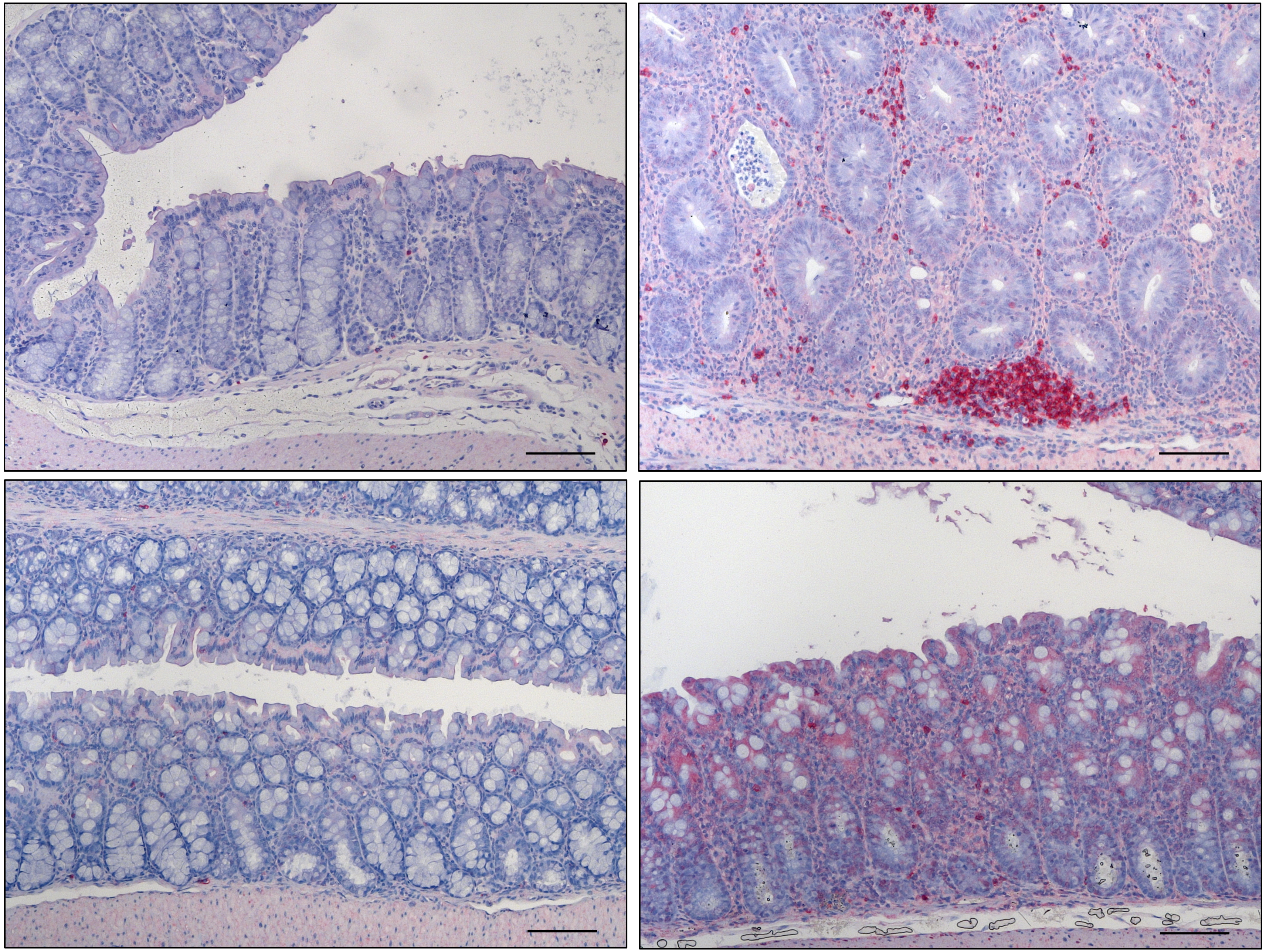

E. coli
TLR4-/-
IL10-/-

C. coli
TLR4-/-
IL10-/-
