# Supplementary figures and images for: Toll-Like Receptor-4 Is Involved in Mediating Intestinal and Extra-Intestinal Inflammation in Campylobacter coli-Infected Secondary Abiotic IL-10−/− Mice
Source: Microorganisms. 2020 Nov 27;8(12):1882. doi: 10.3390/microorganisms8121882 (PMC7761268; doi:10.3390/microorganisms8121882)

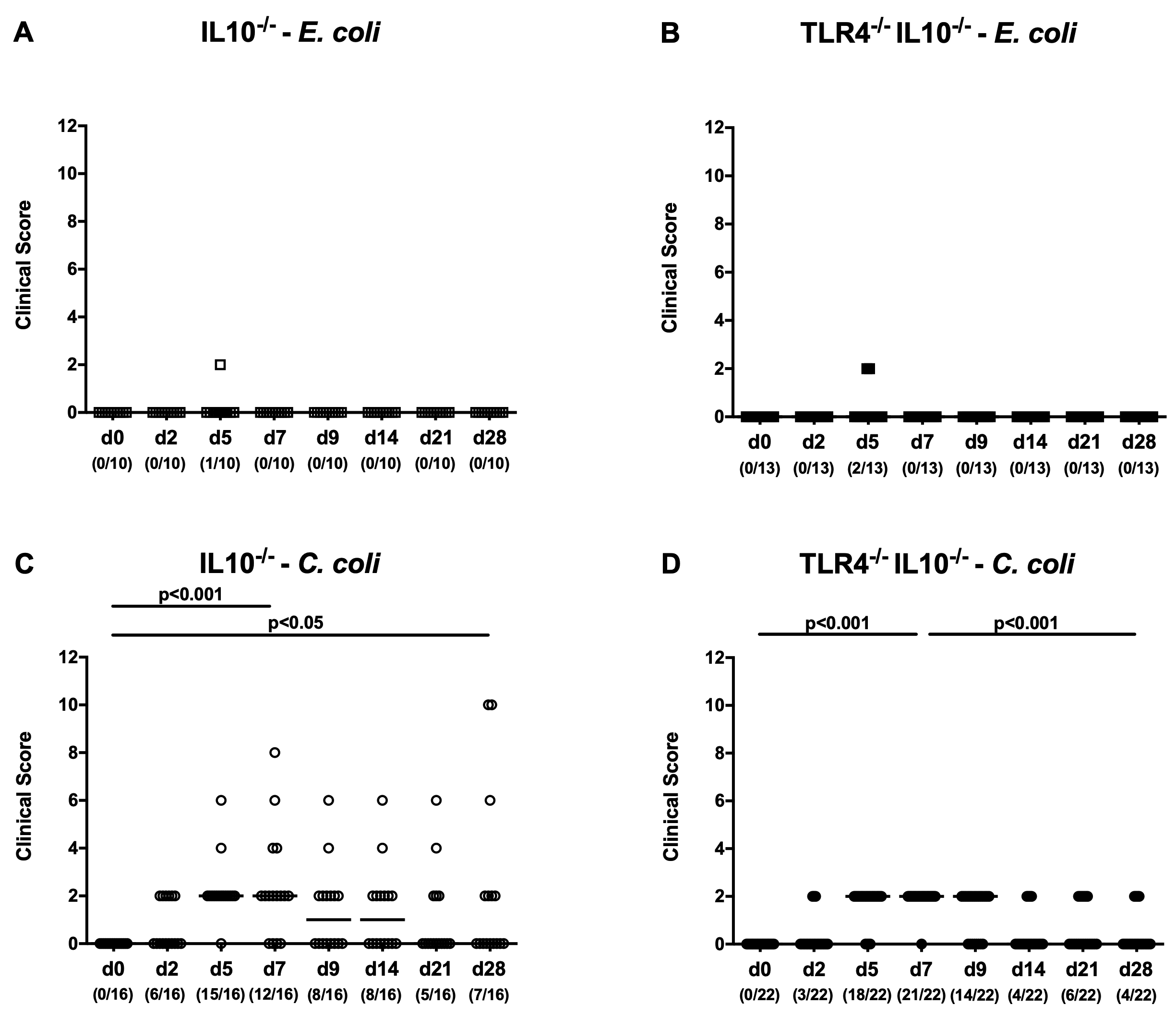

Supplement: Supplementary file 1 [file microorganisms-08-01882-s001.zip › Supplementary_FigS1_GNOTO_IL10_TLR4_13.01.20.tiff]
